# Notes on Preparations for the Sick

**Published:** 1887-03

**Authors:** 


### Notes on Preparations for the Sick.

---

**Benger's Preparations of the Natural Digestive Ferments.** Mottershead & Co., Manchester.

The profession is now keenly alive to the value of the process of artificial digestion in the preparation of foods for the invalid; and in no respect has treatment undergone more improvement in recent years than with regard to the use of peptonised foods in cases of gastric disease, or in cases of great prostration, where the digestive power of the stomach is in abeyance. The name of Mr. Benger has been closely associated with that of Sir William Roberts in connection with the introduction of peptonising agents, and to them jointly the physician and the invalid are infinitely indebted for the potent aids to recovery which their work has given. The text books on stomach disease need to be entirely re-written; and it is not too much to say that peptonising materials are by far the most important drugs in the treatment of ulcer and other stomach affections. We have not seen statistics on this point; but it would be of interest to contrast the per-centage recoveries of cases of gastric ulcer under present treatment and before the process of artificial digestion had come into daily use. Our own experience is that recovery from gastric ulcer is now the rule, and that the persistence of ulcer for years, in spite of treatment, is likely to be the exception.

Another fact bearing on this subject is the abolition of wet-nurses. The custom of endeavouring to preserve the life of a weakly child of well-to-do parents, by risking that of some other presumably less valuable life—apart from the moral and social aspect of the question—now has no rational medical foundation, inasmuch as artificial foods can be prepared which will be more easily digested than the mother's milk, and will answer the purpose far better and with less risk to the child than is entailed by a wet-nurse.

**Liquor Pancreaticus (Benger)** is now so well known that it needs no commendation. As it was the first, so it remains one of the best, means by which milk can be predigested, when the casein cannot be naturally transformed into peptones by natural means and without risk to the organs diseased. Its great utility depends upon the facility with which it can be used for the preparation of partially digested foods without materially impairing their natural flavour. It is true that if digestion be carried too far, the product becomes bitter and nauseous; but the action should be stopped by boiling before the final stages of pancreatic digestion are reached, and the intermediate condition—full solution of the insoluble casein, with an absence of bitter nitrogenous products—can be obtained by attention to the instructions for use which always accompany the fluid. As regards the necessity for boiling, this may be dispensed with when the peptonised product is intended for immediate use, or when intended for rectal alimentation, as in this case a bitter taste will not be of consequence; but if the product be intended to last for twelve hours or longer, at the temperature of the air in warm weather, boiling becomes a real necessity.

**Benger's Peptonising Powders.**—We have found these powders to be most efficient and convenient peptonising agents. They are perfectly soluble, colourless, and odourless; and each powder will completely peptonise one pint of milk. Where the milk is only required at long intervals, we have found it convenient to prepare half a pint at one time, and eliminate the process of boiling, the milk being prepared just when required for immediate use; but, of course, if peptonised, or partially peptonised, milk has to be kept, it must be boiled, whatever peptonising agent is used, or the process will go on till the product becomes disagreeable.

We have had no complaint from patients as to the taste of the milk after the use of these powders; on the other hand, one patient, who has tried all the usual methods, states that with Benger's powders, the taste is practically unchanged, and hence she prefers them to the other methods. She has also observed that now and then vomited matters contained curdled casein when only peptonised milk was taken; but this has not been observed when the milk had been peptonised with the Benger's powders. This patient peptonises half a pint with half a powder, and takes it whilst hot: she prefers to take it freshly prepared and warm.

**Liquor Pepticus (Benger).**—This preparation we have found to be and to do all which its maker claims for it. It is a concentrated and exceedingly active fluid pepsine, acidulated slightly; and it keeps well.

For internal administration it is one of the most active aids to digestion in the stomach. For experimental purposes in the laboratory we have always found it to be exceedingly active, and of course it is likely to be still more efficient when acting under normal thermic and mechanical conditions inside the stomach.

**Benger's Peptonised Beef Jelly.**—An agreeable, partially digested food, containing more real nutriment than the ordinary meat extracts, readily soluble, and likely to be absorbed easily and quickly. Taken cold as a jelly it is very convenient for the bedside, as it requires no preparation, and should be especially valuable for old people and invalids who cannot go many hours without nourishment, and require something at intervals through the night or in early morning. Dissolved in hot water, it constitutes an easily made and highly nutritious beef-tea restorative.

**Benger's Peptonised Chicken Jelly** is an elegant variety of food, physiologically similar to the above, but with a different flavour, and likely to be useful to patients who require frequent changes in their dietetic programme.

**Benger's Food,** for Infants, Children, and Invalids.—This preparation is a cooked and pancreatised farinaceous food. It is superior to malted foods for the following reasons: when mixed with warm milk, or milk and water, the pancreatic ferments not only render the starchy matter soluble, but partly digest the casein of the milk, so that hard, indigestible masses of curd cannot form in the stomach. We have given it in many cases, and have found it to fulfil all the purposes for which it has been devised. It is pleasant to the palate, and is one of the most acceptable and useful preparations for patients in acute diseases where solid food is prohibited—typhoid fever, for instance; as also where a variety from peptonised milk is needed, as in cases of ulcer and other chronic gastric diseases.

---

**"Lion" Food Delicacies.** EDGE BROTHERS, LIMITED, London.

We have received three of these preparations, which we have tried and found excellent. The Beef Essence and Chicken Essence are most inviting in appearance, mounted in air-tight, elegant glass bottles, showing the contents bright and clear, and equally agreeable to the taste. They are evidently prepared with the greatest care, the fatty matter having been removed by filtration through paper, leaving the soluble nitrogenous constituents of the colour and consistence of calf's foot jelly. They are best taken cold, and are most convenient and efficient restoratives in all cases of prostration where more solid nutriment cannot be taken. We have found them to be very useful in cases of gastric disease where even peptonised milk could only be taken in limited quantity.

The Beef Tea Jelly is also mounted in glass bottles, and is very convenient for the ready preparation of beef tea by diluting with hot water. It is made from the best portions of beef, all glutinous and sinewy tissue being carefully rejected: it is rich in nutritive matters, potash salts, and phosphates; and has a flavour resembling the best home-made beef tea.

The "Lion Brand" preparations form a most useful addition to the resources of the physician in the management of the sick, and we can recommend them strongly to the notice of those who have not yet begun to appreciate their advantages.

---

**Coca: Non-alcoholic Table Water.** J. SCHWEPPE & CO., LIMITED, London.

We have received a sample of this beverage, and also of a Purified Essence of Coca Leaf, each fluid ounce of which contains the active principles of one ounce by weight of the leaves of the Erythroxylon Coca.

The uses of Cocaine are now generally recognised, and seem likely to steadily increase. It is no novelty that it should be employed as a beverage, inasmuch as the Indians of Peru have so used it from times of antiquity. We doubt if this combination is likely to become a popular drink, inasmuch as those who require a non-alcoholic effervescing water will probably prefer Apollinaris; and those who require a drug will dilute the essence for themselves, either with Seltzer or plain water.

---

**Malt Extract.** M. HOFF, Hamburg and London.

The Hamburg Hoff's Malt Extract, a fluid described as "the beverage of health," is said to be a concentrated diet peculiarly suitable to convalescents and invalids. It is also considered to be a superior and palatable table beverage and tonic, more especially applicable to abstainers from all intoxicants. It is put up in bottles, and in appearance resembles stout: it is intended to be taken in its natural state with the food, in quantity amounting to one bottle daily.

It appears to have obtained a world-wide reputation, and its manufacturers claim for it a great variety of uses in many diseases, ranging from mild dyspepsia up to tubercular phthisis; in the latter case, its regular use is said to prolong life for ten or even twenty years. Such statements are difficult to prove, and equally so to disprove: we are not inclined to attempt the latter, as the chemical composition of the compound shows it to have a decided nutritive value, both as an aid to digestion and in supplying materials for blood making.

We think that the taste for stout is always acquired; nevertheless, it is a popular drink. In the same way it would be easy to acquire a taste for this useful beverage, and accordingly it might be as popular as stout. As yet, we have not found anyone naturally educated up to its standard of taste: might not this standard be more in harmony with the uneducated palate?

---

**Aesculap.** The Aesculap Bitter Water Co., Limited, London.

We have received samples of this, one of the best known and most popular saline aperients. Its purgative properties are due to the sulphates of soda and magnesia, which are materially modified by the presence of a large proportion of bicarbonate of soda. It accordingly serves the double purpose of neutralising the acid of the gastric fluids, and then acting as an efficient stimulant to intestinal secretion, culminating in the copious discharge of watery evacuations. We have used it frequently, and have found it to be one of the most efficient waters of its kind. Every precaution is taken to secure freedom from organic impurity, and its comparative tastelessness gives it a decided superiority.

---

**Pepsina Liquida (Schacht).** Giles, Schacht, & Co., Clifton, Bristol.

This preparation has an extraordinary solvent energy on comminuted egg albumen. We have seen the product obtained by digesting in a water-bath, at 130° F., of one thousand grains of egg albumen in one drachm of the liquid pepsine, with twenty-eight minims of hydrochloric acid and six ounces of water. The solution is clear, and devoid of disagreeable odour: it gives no precipitate on boiling, and none with acetic acid; but precipitates abundantly on neutralisation, and with nitric acid. The precipitate with picric acid in Ebstein's albuminometer indicates about one per cent. of dried albumen in solution. On testing for peptones with Fehling's solution, we obtained the biuret reaction; and on dialysing the solution for six hours we found indications of peptone in the dialysate, as shown by a slight precipitation with tannic acid, also with picric acid, and with potassio-mercuric iodide.

These tests show that the product of the action of the liquid on egg albumen is an exact imitation of the first stages of gastric digestion—*the formation of a solution of acid albumen*—and that little of the albumen is transformed into peptone. The molecule has clearly not become so transformed as to be capable of ready dialysis: little albuminous matter was found outside the dialyser, whereas the fluid after dialysis still gave a precipitate with picric acid, indicating the presence of ·68 °/₀ of albumen in solution.

We must congratulate Mr. Schacht on his success in the preparation of a fluid which has so powerful a solvent action on albuminous foods, and is at the same time bright, inodorous, palatable, and inexpensive.

---

**Compound Syrup of the Hypophosphites.** LORIMER & Co., London.

This is a syrup composed of the hypophosphites of lime, sodium, potassium, iron, manganese, quinine, and strychnia, combined in a clear solution, which is neither acid nor alkaline; is colourless, odourless, and agreeable to the palate. A syrup of this composition, and with no disagreeable qualities, must have a very wide range of utility. The physician need not hesitate to prescribe a compound of which the composition is indicated on the label; and the number of medicinal substances mentioned there will suggest the idea that a corresponding large number of diseases are likely to be benefited by the use of one or other of the components. We have, however, little faith in the selective power of individual ingredients for particular tissues or organs. The compound, as a whole, has a composition which, on theoretical grounds, should be of great value in many varieties of defective nutrition, and which is found by the test of experience to perform everything which can reasonably be expected of it. We commend this syrup to those who have not used it, in full confidence that it will not disappoint them.

